# Help‐seeking anxiety, psychological distress, and suicidal ideation among medical and nursing students: A cross‐sectional study

**DOI:** 10.1002/pcn5.70353

**Published:** 2026-06-04

**Authors:** Kentaro Kawabe, Yu Matsumoto, Yasutaka Ihara, Fumie Horiuchi

**Affiliations:** ^1^ Department of Child Psychiatry Ehime University Graduate School of Medicine Toon Ehime Japan; ^2^ Department of Neuropsychiatry Ehime University Graduate School of Medicine Toon Ehime Japan; ^3^ Clinical Research Promotion Unit Clinical Therapeutic Trial Center, Ehime University Hospital Toon Ehime Japan

**Keywords:** help‐seeking, psychological distress, suicidal ideation, suicide prevention, university students

## Abstract

**Aim:**

Suicide among young adults has become a serious public health concern. In Japan, suicide is a leading cause of death among adolescents and young adults. Suicidal ideation is a clinically meaningful psychological burden among young adults. However, the associations of suicidal ideation with help‐seeking anxiety, psychological distress, and subjective well‐being among healthcare students have not been explored sufficiently. This study aimed to examine the cross‐sectional associations of help‐seeking anxiety, psychological distress, and subjective well‐being with suicidal ideation among first‐year healthcare students.

**Methods:**

A cross‐sectional web‐based survey was conducted among 142 first‐year medical and nursing university students in Japan. Suicidal ideation was assessed using the Suicidal Ideation Questionnaire, psychological distress was assessed using the Kessler Psychological Distress Scale, subjective well‐being was assessed using the World Health Organization 5‐item Well‐Being Index (WHO‐5), and help‐seeking anxiety was assessed using the Thoughts About Therapy Survey. Associations among help‐seeking anxiety, psychological distress, subjective well‐being, and suicidal ideation were examined using Spearman's correlation analysis and multivariable regression analyses.

**Results:**

Higher help‐seeking anxiety was associated with higher levels of suicidal ideation and psychological distress. Psychological distress was also associated with suicidal ideation, whereas subjective well‐being was not clearly associated with suicidal ideation. In the multivariable regression analyses, help‐seeking anxiety remained associated with suicidal ideation after accounting for psychological distress and subjective well‐being. Additionally, most students reported having someone to consult when experiencing distress; however, awareness of university mental health services was limited.

**Conclusion:**

These findings highlight suicidal ideation as a clinically meaningful psychological burden among first‐year healthcare students and suggest that psychological distress and help‐seeking anxiety are associated with suicidal ideation.

## Graphical abstract

In a cross‐sectional study of first‐year healthcare students in Japan, help‐seeking anxiety and psychological distress were associated with suicidal ideation. These findings highlight suicidal ideation as a clinically meaningful psychological burden and suggest that help‐seeking anxiety may reflect perceived barriers to accessing professional support.

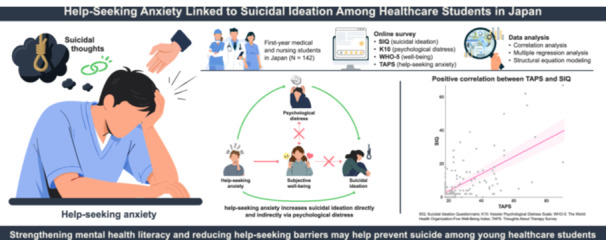

## INTRODUCTION

Suicide is one of the leading causes of death worldwide and is a serious public health issue among young people.^1^ According to vital statistics reported by the Ministry of Health, Labor, and Welfare, suicide has remained the leading cause of death among individuals aged 15–29 years in Japan for many years. Therefore, suicide prevention is critical during adolescence and early adulthood. Furthermore, reports based on suicide statistics compiled by the National Police Agency indicate that the provisional number of suicides among elementary, junior high, and high school students reached 532 in 2025, the highest number recorded since the statistics began in 1980.^2^ While the total number of suicides in Japan has generally declined in recent years, the number of suicides among individuals aged 19 years or younger has continued to increase. These trends underscore the need to prioritize suicide prevention efforts targeting adolescence and early adulthood in Japan.

Furthermore, identifying early warning signs and risk factors is essential in suicide prevention strategies. Suicidal ideation and self‐injurious behavior are among the strongest predictors of future suicide attempts and deaths.^3^ Adolescence and early adulthood are developmental periods characterized by heightened impulsivity, immature emotion regulation, and unstable interpersonal relationships, which may increase vulnerability to suicidal behaviors.^4^ Therefore, early detection of suicidal ideation and intervention are critical components of suicide prevention. In Japan, national suicide prevention policies emphasize the importance of strengthening measures targeting children and young people. While suicide among elementary and high school students has been steadily increasing, schools are trying to care for the mental health of students with the help of teachers, school nurses, and school counselors. In addition, community‐based services and child and family support centers offer consultation and assistance to both students and their families. However, the transition from high school to university is a major life change. Students entering university, particularly those who relocate away from their families, may face multiple stressors, including disruptions in their daily routines, increased feelings of isolation, financial concerns, and the need to establish new social relationships. In this context, psychological barriers to seeking help, such as help‐seeking anxiety, are important factors influencing mental health outcomes. Help‐seeking anxiety refers to concerns or resistance toward accessing psychological support, including stigma, fear of negative evaluation, and doubts about the effectiveness of professional services.^5^ Such barriers may delay or prevent appropriate care, thereby increasing the risk of suicidal ideation among university students.^6^

These issues may be particularly relevant among healthcare students, such as medical and nursing students. Previous studies have reported that medical students experience high levels of psychological distress and anxiety, partly due to academic pressure and demanding training environments.^7^ A recent study reported that anxiety symptoms are common among medical students and that multiple barriers hinder their help‐seeking behaviors.^8^ Medical students may avoid seeking mental health support due to concerns about stigma and potential negative impact on their academic and professional records.^9^ Furthermore, high prevalence rates of depression and anxiety have also been reported among first‐year healthcare students.^10^ Thus, understanding help‐seeking anxiety in this population is particularly important for developing effective suicide prevention strategies.

Mental health support in universities is typically provided through campus health or counseling centers; however, its utilization remains relatively low. A large‐scale survey of university students in the United States reported that many students with clinically significant depressive symptoms did not receive professional mental healthcare.^11^ In Japan, it has also been reported that approximately 20% of university students who died by suicide had previously been in contact with university health centers.^12^ As educational systems, family structures, access to mental health services, and cultural attitudes toward mental illness differ between Japan and Western countries, findings from overseas studies cannot be directly applied to the Japanese context. Therefore, accumulating evidence that reflects the sociocultural context of Japan is essential. Based on these considerations, this study focused on first‐year medical and nursing students and aimed to examine the cross‐sectional associations of help‐seeking anxiety, psychological distress, and subjective well‐being with suicidal ideation.

## METHODS

### Participants

This study employed a cross‐sectional design using a web‐based questionnaire. The study population consisted of first‐year students enrolled in the Faculty of Medicine at the University in 2025. Participation was voluntary, and students who provided informed consent were included in the analysis. Students who did not provide consent were excluded from the study. This study was conducted between October and December 2025.

### Procedure and ethical considerations

Data were collected using an anonymous web‐based questionnaire created with Microsoft Forms. The researcher coordinated with the academic affairs offices, provided a brief explanation of the study, and invited them to participate in the survey during scheduled class sessions. After reading the study information, participants indicated their consent by selecting an agreement button on the web form before accessing the questionnaire. Participation was entirely voluntary, and students were informed that they could decline or withdraw at any time without any disadvantage. This study was approved by the Institutional Review Board (IRB) of Ehime University Graduate School of Medicine (IRB No. 2509007).

### Instruments

#### Background characteristics

Participants reported demographic information, including age, sex, place of birth, faculty affiliation, the presence of someone they could consult, and knowledge of the university health center.

#### Suicidal ideation

Suicidal ideation was assessed using the 15‐item Suicidal Ideation Questionnaire (SIQ). SIQ is a widely used self‐report measure that evaluates the frequency and severity of suicidal thoughts among adolescents and young adults; it has demonstrated good reliability and validity. Higher scores indicate greater severity of suicidal ideation.^13^ Data on the reliability and validity of the SIQ have been reported in clinical and school‐based samples.^14^

#### Psychological distress

Psychological distress during the past 4 weeks was assessed using the Kessler Psychological Distress Scale (K10). Furthermore, K10 is suitable for assessing morbidity in the population and appropriate for clinical practice.^15^ Additionally, K10 consists of 10 items scored on a five‐point scale, with total scores ranging from 10 to 50. Higher scores indicate greater psychological distress, and a score of 25 or higher has been suggested as the cutoff for severe distress. This scale has demonstrated high reliability and broad applicability across populations.^16^

#### Subjective well‐being

Subjective well‐being was measured using the World Health Organization 5‐item Well‐Being Index (WHO‐5). Furthermore, WHO‐5 is among the most widely used questionnaires, and it consists of five items assessing subjective psychological well‐being over the previous 2 weeks.^17^ Raw scores range from 0 to 25, with higher scores indicating better well‐being. The Japanese version of the WHO‐5 has demonstrated good reliability and validity.^18^, ^19^

#### Help‐seeking anxiety

Barriers to help‐seeking were assessed using the Thoughts About Therapy Survey (TAPS).^20^ The TAPS measures concerns that may inhibit help‐seeking for psychological support. This study used the three‐factor model validated among Japanese university students, consisting of concerns about stigma, coercion, and counselor responsiveness.^21^ Items were rated on a five‐point scale, with higher scores indicating greater help‐seeking anxiety.

### Statistical analysis

Descriptive statistics were calculated for background characteristics and each scale. Continuous variables were summarized using the number of observations, mean, standard deviation, median, and interquartile range, whereas categorical variables were summarized as counts and percentages. Spearman's correlation analysis was conducted to examine the association between SIQ and TAPS scores.

Multiple regression analyses were performed with the SIQ score as the dependent variable and the TAPS score as the primary independent variable. In the primary regression model, we adjusted for background characteristics to estimate the background‐adjusted total association between TAPS and SIQ. Because K10 and WHO‐5 were conceptualized as potential intermediate variables in the hypothesized structural equation modeling (SEM), they were not included in the primary regression model. In a secondary conditional regression model, K10 and WHO‐5 scores were additionally included to examine whether TAPS remained associated with SIQ after accounting for psychological distress and subjective well‐being.

To further explore the hypothesized statistical relationships among TAPS, K10, WHO‐5, and SIQ, an exploratory path analysis using SEM was conducted. Paths were specified from TAPS to K10 and WHO‐5, and from TAPS, K10, and WHO‐5 to SIQ. This model was used to estimate path coefficients and path‐specific product‐term estimates under the hypothesized statistical model. Because this study used a cross‐sectional design, these estimates were interpreted as exploratory statistical associations rather than evidence supporting temporal ordering, mediation mechanisms, or causal pathways.

Missing values in the regression analyses were handled using multiple imputation by chained equations with 10 imputed datasets, and estimates were combined using Rubin's rules. Statistical significance was set at a two‐sided *p* value of <0.05. Analyses were performed using R version 4.4.2 (R Foundation for Statistical Computing, Vienna, Austria).

## RESULTS

**Figure 1:**
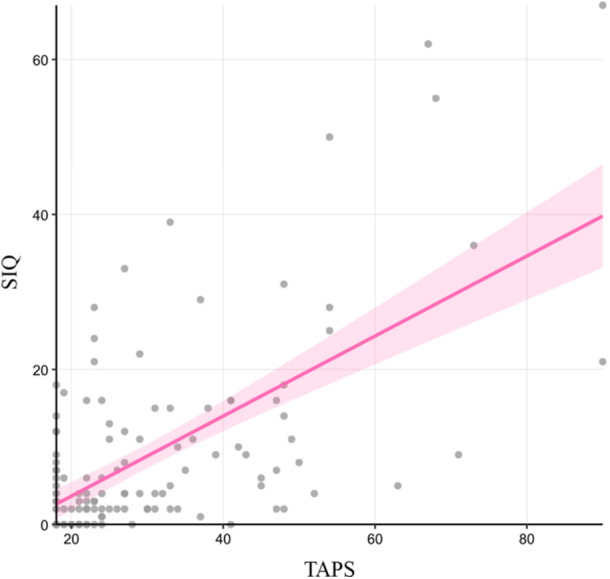
Correlation between help‐seeking anxiety (Thoughts About Therapy Survey [TAPS]) and suicidal ideation (Suicidal Ideation Questionnaire [SIQ]). A significant positive correlation was observed (Spearman's *r* = 0.543; 95% confidence interval [CI], 0.42–0.65; *p* < 0.001).

**Figure 2:**
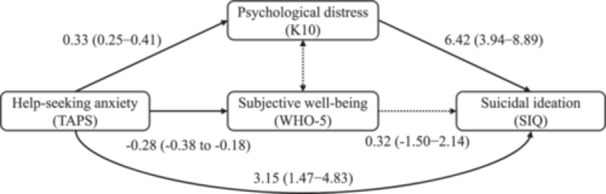
Exploratory structural equation model of the hypothesized statistical relationships among help‐seeking anxiety (Thoughts About Therapy Survey [TAPS]), psychological distress (Kessler Psychological Distress Scale [K10]), subjective well‐being (World Health Organization 5‐item Well‐Being Index [WHO‐5]), and suicidal ideation (Suicidal Ideation Questionnaire [SIQ]). Values represent unstandardized path coefficients with 95% confidence intervals. This model was used only for hypothesis‐generating purposes. Because the model showed poor global fit, the paths should not be interpreted as evidence supporting mediation mechanisms, temporal ordering, or causal pathways.

A total of 144 students initially registered to participate in this study. However, two students were excluded as informed consent was not obtained, leaving 142 participants for the final analysis. The background characteristics of the participants are listed in Table 1. Moreover, 83 participants (58.5%) were enrolled in the medical program and 59 (41.5%) in the nursing program. Regarding sex, 103 participants (72.5%) were female, and 37 (26.1%) were male, while two participants (1.4%) did not report their sex. The majority of participants were from Ehime Prefecture (*n* = 88, 62.0%). Most students reported having someone they could consult when experiencing psychological distress (*n* = 133, 93.7%). In contrast, only 39 students (27.5%) reported being aware of the university health service. Descriptive statistics for each psychological measure are presented in Table 2. Spearman's correlation analysis revealed a significant positive correlation between TAPS and SIQ scores (*r* = 0.543; 95% confidence interval [CI], 0.42–0.65; *p* < 0.001) (see Figure 1). In the primary regression model adjusted for background characteristics, a higher TAPS score was associated with a higher SIQ score (estimate per 10‐point increase, 4.76; 95% CI, 3.59–5.93; *p* < 0.001). In the secondary conditional model additionally adjusted for K10 and WHO‐5 scores, TAPS remained associated with SIQ (estimate per 10‐point increase, 2.83; 95% CI, 1.62–4.04; *p* < 0.001). K10 was also associated with SIQ in the secondary conditional model, whereas WHO‐5 and background characteristics were not clearly associated with SIQ (Table 3). To further examine the hypothesized statistical relationships among TAPS, K10, WHO‐5, and SIQ, an exploratory SEM was conducted (Figure 2 and Table 4). The model showed poor global fit: *χ* ^2^ = 25.85, df = 1, *p* < 0.001, comparative fit index (CFI) = 0.886, tucker–lewis index (TLI) = 0.315, root mean square error of approximation (RMSEA) = 0.418 (95% CI, 0.320–0.525), and standardized root mean square residual (SRMR) = 0.119. Therefore, the SEM results were not interpreted as evidence supporting the hypothesized structural model or mediation mechanisms. Within this poorly fitting exploratory model, TAPS was statistically associated with K10, WHO‐5, and SIQ, and K10 was statistically associated with SIQ, whereas WHO‐5 was not clearly associated with SIQ. Under the specified model, the path‐specific estimate corresponding to the TAPS–K10–SIQ path was observed, whereas that corresponding to the TAPS–WHO‐5–SIQ path was not clearly observed. These findings should be regarded only as hypothesis‐generating statistical associations and should not be interpreted as evidence of temporal ordering, mediation mechanisms, or causal pathways.

**Table 1:** Characteristics of the participants (*N* = 142).

Variable	Category	*N* (%)
Program	Medical	83 (58.5)
	Nursing	59 (41.5)
Sex	Male	37 (26.1)
	Female	103 (72.5)
	Not reported	2 (1.4)
Age, years	18	32 (22.5)
	19	80 (56.3)
	20	23 (16.2)
	Over 21	7 (4.9)
Place of birth	University prefecture	88 (62.0)
	Neighboring prefectures	4 (2.8)
	Other areas in Western Japan	37 (26.1)
	Eastern Japan	13 (9.2)
Someone to consult when distressed	Yes	133 (93.7)
	No	9 (6.3)
Awareness of the university health service	Yes	39 (27.5)
	No	103 (72.5)

**Table 2:** Descriptive statistics of psychological measures.

Measure	Mean	SD	Median	IQR
WHO‐5	16.19	4.90	17	13–20
K10	19.78	8.67	18	12–24
SIQ	8.22	11.99	4	0–11
TAPS	28.83	14.66	23	18–33
Stigma	16.30	8.28	13	10–20
Coercion concern	7.84	4.49	5	5–9
Counselor responsiveness concerns	4.70	2.72	3	3–6

Abbreviations: IQR, interquartile range; K10, Kessler Psychological Distress Scale; SD, standard deviation; SIQ, Suicidal Ideation Questionnaire; TAPS, Thoughts About Therapy Survey; WHO‐5, World Health Organization 5‐item Well‐Being Index.

**Table 3:** Association between help‐seeking anxiety and suicidal ideation.

Variable	Model 1		Model 2	
	Estimate (95% CI)	*p*	Estimate (95% CI)	*p*
TAPS, per 10‐point increase	4.76 (3.59, 5.93)	<0.001	2.83 (1.62, 4.04)	<0.001
K10, per 10‐point increase			6.49 (4.17, 8.81)	<0.001
WHO‐5, per 5‐point increase			0.22 (−1.65, 2.09)	0.819
Program, ref; medical	2.46 (−1.31, 6.23)	0.199	1.36 (−2.00, 4.71)	0.426
Male, ref; female	1.95 (−2.06, 5.96)	0.337	1.80 (−1.73, 5.33)	0.314
Age 19, ref; 18	0.38 (−3.54, 4.30)	0.847	2.16 (−1.35, 5.67)	0.225
Age over 20, ref; 18	1.09 (−4.06, 6.24)	0.676	3.20 (−1.39, 7.80)	0.170
Place of birth, ref; university prefecture	1.78 (−1.61, 5.17)	0.301	1.62 (−1.38, 4.62)	0.288
Someone to consult when distressed, ref; no	−6.05 (−12.81, 0.72)	0.080	−2.61 (−8.69, 3.47)	0.398
Awareness of the university health service, ref; no	0.91 (−2.73, 4.55)	0.622	1.20 (−2.02, 4.42)	0.464

*Note*: Model 1 adjusted for background characteristics, including faculty affiliation, sex, age, place of birth, the presence of someone the student could consult, and awareness of the university health center. Model 2 additionally adjusted for K10 and WHO‐5 scores. Because K10 and WHO‐5 were included in the hypothesized SEM as variables statistically related to both TAPS and Suicidal Ideation Questionnaire (SIQ), Model 2 should be interpreted as a conditional association model rather than a model estimating the total association between TAPS and SIQ.

Abbreviations: CI, confidence interval; K10, Kessler Psychological Distress Scale; TAPS, Thoughts About Therapy Survey; WHO‐5, World Health Organization 5‐item Well‐Being Index.

**Table 4:** Exploratory path coefficients and path‐specific estimates under the prespecified structural equation model.

	Estimate (95% CI)	*p*
*Path coefficients*		
K10 ← TAPS	0.33 (0.25–0.41)	<0.001
WHO‐5 ← TAPS	−0.28 (−0.38 to −0.18)	<0.001
SIQ ← TAPS	3.15 (1.47–4.83)	<0.001
SIQ ← K10	6.42 (3.94–8.89)	<0.001
SIQ ← WHO‐5	0.32 (−1.50 to 2.14)	0.733
*Path‐specific and total estimates*		
TAPS–K10–SIQ path‐specific estimate	2.10 (1.02–3.19)	<0.001
TAPS–WHO‐5–SIQ path‐specific estimate	−0.09 (−0.59 to 0.42)	0.734
Total path‐specific estimate	2.02 (1.06–2.97)	<0.001
Total association under the specified model	5.16 (3.15–7.17)	<0.001

*Note*: The SEM was conducted as an exploratory, hypothesis‐generating analysis. Because the model showed poor global fit, the path coefficients and path‐specific estimates should not be interpreted as evidence supporting the specified structural model, mediation mechanisms, temporal ordering, or causal pathways.

Abbreviations: CI, confidence interval; K10, Kessler Psychological Distress Scale; SIQ, Suicidal Ideation Questionnaire; TAPS, Thoughts About Therapy Survey; WHO‐5, World Health Organization 5‐item Well‐Being Index.

## DISCUSSION

This study examined the associations among help‐seeking anxiety, psychological distress, subjective well‐being, and suicidal ideation among university students. The results showed that higher help‐seeking anxiety was associated with higher levels of suicidal ideation in the background‐adjusted regression model. In the model additionally adjusted for psychological distress and subjective well‐being, help‐seeking anxiety remained associated with suicidal ideation, and psychological distress was also associated with suicidal ideation. These findings suggest that help‐seeking anxiety and psychological distress were both associated with suicidal ideation in this study population. However, because of the cross‐sectional design, these findings should be interpreted as cross‐sectional statistical associations and not as evidence of causal relationships or temporal ordering.

Previous studies have reported that although the prevalence of mental health problems is relatively high among university students, stigma and psychological resistance toward seeking help often act as barriers to the use of mental health services.^22^ In addition, self‐reliance and self‐stigma, such as the belief that personal problems should be solved independently, have been identified as common reasons why university students do not seek professional support.^23^ This issue may be more pronounced among medical and nursing students, where stigma and academic or career concerns discourage help‐seeking.^7^, ^9^ These psychological barriers may reflect perceived concerns about accessing professional support. In the present study, help‐seeking anxiety was associated with both psychological distress and suicidal ideation; however, actual help‐seeking behavior was not assessed.

In the regression analyses, psychological distress was associated with suicidal ideation, whereas subjective well‐being was not clearly associated with suicidal ideation. These findings suggest that psychological distress may be more closely related to suicidal ideation than subjective well‐being in this study population. However, because this study was cross‐sectional, this interpretation should be regarded as exploratory and not as evidence of a temporal or causal relationship. Previous studies have reported that psychological distress is strongly associated with suicidal thoughts and behaviors among young people.^3^ Therefore, psychological distress may be an important factor to consider when characterizing suicidal ideation among university populations, especially among medical and nursing students experiencing high levels of distress.^24^

Another finding of this study was the discrepancy between the availability of informal support from friends or family and awareness of campus‐based mental health services. Although most students reported having someone they could consult when experiencing psychological distress, few students were aware of the university health service center. This finding suggests that students may rely primarily on informal sources of support, while their awareness of professional mental health services at the university remains limited. Although informal support can be beneficial, it may be insufficient for students with serious psychological difficulties. Therefore, improving students' awareness of and access to professional support services in universities may be important for supporting students experiencing psychological distress or suicidal ideation. Previous studies have suggested that help‐seeking behavior among university students is complex.^25^ For example, Kearns et al.^22^ reported that students with a stronger sense of belonging to their university were paradoxically less likely to use on‐campus counseling services, highlighting the importance of reducing psychological barriers to help‐seeking. Notably, several studies have reported that many students experiencing suicidal ideation do not access professional mental health services, underscoring the importance of understanding perceived barriers and psychological concerns related to seeking support.^6^, ^26^ In Japan, some universities have implemented the Crisis‐management, Anti‐stigma, and Mental Health Literacy Program for University Students (CAMPUS), a suicide prevention program targeting university students. Educational programs focusing on mental health literacy and stigma reduction have been reported to improve help‐seeking intentions and may be associated with lower levels of suicidal ideation among students.^27^ Such approaches may be relevant to future efforts to address psychological barriers and perceived concerns about seeking support among university populations.

This study has several limitations. First, this study used a cross‐sectional design; therefore, causal relationships and the temporal ordering among help‐seeking anxiety, psychological distress, subjective well‐being, and suicidal ideation could not be established. The findings should not be interpreted as evidence that modifying help‐seeking anxiety or psychological distress would reduce suicidal ideation. In addition, this study was not designed to develop or validate a suicide risk prediction model, and the findings should not be used for individual‐level risk assessment or risk stratification, particularly given the known limitations of suicide risk prediction models.^28^ The exploratory SEM was conducted only as a hypothesis‐generating analysis based on a prespecified conceptual model. Because the SEM showed poor global fit and the cross‐sectional design precluded assessment of temporal ordering, it should not be interpreted as evidence supporting the specified structural model, mediation mechanisms, or causal pathways, consistent with methodological concerns regarding mediation analyses using cross‐sectional data.^29^, ^30^ Longitudinal studies are needed to clarify temporal relationships among these factors and to examine whether changes in help‐seeking anxiety or psychological distress are associated with subsequent changes in help‐seeking behavior or suicidal ideation. Second, the data were collected using self‐report questionnaires and may have been influenced by subjective responses. Third, participants were recruited from a single university and were limited to medical and nursing students, which may limit the generalizability of the findings. Fourth, this study assessed help‐seeking anxiety using the TAPS but did not assess actual help‐seeking behavior or use of professional mental health services. Therefore, the findings should be interpreted as associations involving psychological tendencies and perceived barriers rather than behavioral outcomes. Finally, potentially important factors, such as socioeconomic status and history of mental disorders, were not fully assessed.

## CONCLUSION

This study's findings suggest that psychological distress and help‐seeking anxiety are relevant factors associated with suicidal ideation among first‐year healthcare students. Future longitudinal studies are needed to clarify the temporal relationships among help‐seeking anxiety, psychological distress, actual help‐seeking behavior, and suicidal ideation. In addition, creating a university environment in which students feel comfortable accessing professional psychological services may be important for supporting students experiencing psychological distress or suicidal ideation.

## AUTHOR CONTRIBUTIONS

Kentaro Kawabe conceived and designed the study and obtained funding. Kentaro Kawabe and Yu Matsumoto collected the data. Yasutaka Ihara contributed to the study design and statistical analysis and critically reviewed the manuscript for important intellectual content. Fumie Horiuchi provided critical comments that significantly enhanced the final draft. All authors approved the final version of the manuscript.

## CONFLICT OF INTEREST STATEMENT

The authors declare no conflicts of interest.

## ETHICS APPROVAL STATEMENT

The study was approved by the Institutional Review Board of Ehime University Graduate School of Medicine (IRB No. 2509007).

## PATIENT CONSENT STATEMENT

Participants provided their consent by selecting an agreement button on the web form before accessing the questionnaire.

## Data Availability

The data that support the findings of this study are available from the corresponding author upon reasonable request.
